# Extrafloral Nectaries of *Passiflora* Species: Nectar Composition and Secretion, Visitors and Interactions With Herbivores

**DOI:** 10.1002/ece3.74641

**Published:** 2026-11-13

**Authors:** M. T. Amela García, L. Rodríguez‐Planes, G. Gottsberger

**Affiliations:** ^1^ Sistemática y Biología Reproductiva de Plantas Vasculares, Depto. de Biodiversidad y Biología Experimental, Facultad de Ciencias Exactas y Naturales Universidad de Buenos Aires Buenos Aires Argentina; ^2^ Instituto de Micología y Botánica (INMIBO) CONICET—Universidad de Buenos Aires Buenos Aires Argentina; ^3^ Botanical Garden and Herbarium University of Ulm Ulm Germany

**Keywords:** domatia, egg mimics, extrafloral nectar, extrafloral nectar thieves, nectar feeders, *Passiflora*

## Abstract

The extrafloral nectaries of *Passiflora* have different location and morphology; though widespread in this genus, little is known about their functioning and interaction with visitors and, thereafter, with herbivores. The ecology of the extrafloral nectaries of three *Passiflora* species from Argentina was studied with respect to nectary types, nectar chemistry and secretion, as well as occurrence and behavior of nectarivores, folivores, florivores and frugivores in different sites. Sucrose, glucose, fructose and 15 proteinogenic amino acids occurred in all three species. The total sugar amount did not differ between species. The sucrose/hexose proportion was high in all species. The amino acid quantity and quality of *P. caerulea* differed from those of *P. suberosa and P* *. misera* (which belong to a different subgenus), thus amino acid traits might contribute to taxonomic differences. Nectaries in all three species were active only in young leaves. The glands of *P. suberosa* (larger than the ones of the other two species) housing Acari and detritus during the post‐secretory stage might function as incidental domatia. Due to their color, shape and distribution, *P. caerulea* and *P. misera* extrafloral nectaries probably mimic lepidopteran eggs. Nectar production did not vary with varying ant exclusion time in any species but *P. caerulea* secreted again and faster after experimental removal. The assembly of nectar feeders differed between species; co‐occurring specimens of different plant species shared only some consumers, and not all insect taxa were associated with each plant species in every locality. Ants (the most frequent visitors) and wasps seemed to protect *Passiflora* spp. from Heliconiinae caterpillars and Coreids; other hemipterans and flies were nectar thieves.

## Graphical abstract

This paper deals with extrafloral nectaries and nectar description of three species of *Passiflora* included in different subgenera, as well as the assembly and function of the insect visiting taxa. Nectary type and nectar amino acid profile differs between passionvine species but seems to be conservative within the subgenus. The nectary morphology is associated with distinct arthropod usage but no species‐species interaction was detected.

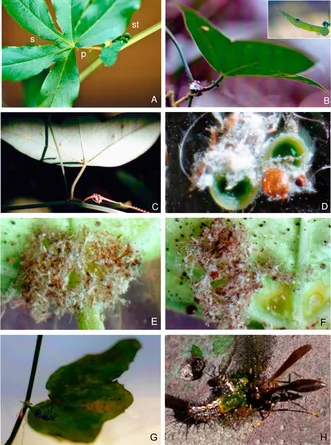

## 1 Introduction

Extrafloral (EF) nectaries are nectar producing glands situated outside the flowers, either in inflorescences axis, fruit exocarps or in vegetative parts, which attract insects that can defend plants from herbivores (Elías 1983). Thus, EF nectaries are part of a protective mutualism, facultative in the majority of the cases (Rico‐Gray and Oliveira 2007), which mediate multitrophic interactions (Becerra 1989; Koptur et al. 2015). They are present in nearly 5000 species (Basnet and Joshee 2026), in almost half of the angiosperm orders (Weber and Keeler 2013), in more than 100 families (Marazzi et al. 2013) and in a few gimnosperms and ferns (Grasso et al. 2015). In angiosperms, they are more common in trees and vines (Miranda et al. 2022), being more effective in defense in vines than in non‐vines (Rico‐Gray and Oliveira 2007), though other factors are more important determinants (see next paragraph). Plant lineages that have evolved EF nectaries exhibit higher diversification rates than non‐EF bearing nectary lineages, so defense mutualisms would enhance plant diversity (Weber and Agrawal 2014). EF nectar thus, in association with the attracted insects, may conform a biotic defense (Moura et al. 2021). Notwithstanding this, EF nectaries often occur on plants additionally provided with chemical or physical defenses (Bentley 1977). Defense type, as well as degree of defense (Blanchard and Holeski 2024), can change through time and space, as, for instance, along leaf lifespan (Calixto et al. 2015). Defenses concentrated in expanding leaves are expected as damage to them is greater than to older ones, mainly in tropical plants (Coley and Barone 1996).

The most common visitors to EF nectaries are ants (519 species after Novais et al. 2025), which have been probed to constitute a defense against herbivores (Keeler 1989; Fiala et al. 1994; Koptur et al. 1998; Katayama and Suzuki 2004; Oliveira et al. 2012; Rosumek et al. 2009; Trager et al. 2010; Calixto et al. 2018). The benefit usually relies on relatively unspecialized interactions (Whalen and Mackay 1988; Miller 2007) that confer different degrees of protection, depending on various factors, such as the intervening ant taxa (Koptur 1984; Gaume and McKey 1999) involving specific levels of aggression and constancy (Miller 2007), plant phenology (Di Giusto et al. 2001), and herbivore (Rudgers and Strauss 2004) as well as ant (Miller 2007) relative densities. Fagundes et al. (2017) have examined the influence of EF nectaries in resource partitioning among sympatric (even not phylogenetically related) taxa and the outcoming protecting ability of the partner ant species depending on the degree of connectivity to the net. Apparently, plants that attract ant mutualists have higher diversification rates than non‐ant associated lineages, but in ants, mutualisms evolve more often in already radiated lineages (Kaur et al. 2019).

Predatory wasps have also been reported as visitors of EF nectaries (Beckman and Stucky 1981; Meira Marques et al. 2005) and probed (Cuatle and Rico‐Gray 2003) or suspected to be (Ward 2002; Alves‐Silva et al. 2012) antiherbivore defenders, though their lesser frequency and constancy may be due to outcompetition by ants (Heil 2015). Parasitoids and insects of other orders (including Diptera, Hemiptera, Lepidoptera) consume EF nectar and may also defend (Heil 2008); anyway, more research about the survey of EF nectary visitors and their effects on the plants, as well as the importance of EF nectar on the nutrition of the consumers and, in consequence, on the structuring of arthropod communities is still lacking (Heil 2015).

Studies concerning EF nectaries and nectar have increased in the last decades (Calixto et al. 2018), peaking in the past ones (Basnet and Joshee 2026). These include nectar composition, secretion pattern (Koptur 1989; Pascal 1993; Lange et al. 2017), visitors (Heil 2008), and their preferences (Blüthgen and Fiedler 2004b), as well as the effects of EF nectar production on ant attractiveness and herbivory (Fagundes et al. 2017; Calixto et al. 2020). Regarding nectar composition, the sugar and amino acid constitution of extrafloral nectar was reviewed by Bentley (1977) and the quantity of each amino acid (Keeler 1980; Inouye and Inouye 1980) and the quality and concentration of sugars and amino acids (Blüthgen et al. 2004) were explored in some taxa. However, studies comparing the compositions of EF nectar within and among species, as well as ones testing the effect of extraction or availability (Kost and Heil 2005), are scarce.

EF nectaries occur in most species of *Passiflora* (Passifloraceae), where these glands show a high variety in location (throughout the leaves) and morphology (Cusset 1965). Descriptions, which have been done from a taxonomic viewpoint (Schnell et al. 1963; Cusset 1965; Deginani 2001), are incomplete and contradictory: that is, for some species the quantity of nectaries per leaf or the size of the nectary is not reported. Even more, different nomenclatures have been applied for the same nectary type. Some studies deal with their anatomy (Roth 1974; López Meruvia et al. 1993; Jáuregui et al. 2002; Vargas‐Silva et al. 2018; Monteiro Macedo et al. 2021; Moraes et al. 2022; Oliveira Silva et al. 2023). EF nectaries are common in gap species, as *Passiflora* species are (usually growing at forest edges and openings), probably because they have a major turnover of leaves than shade‐tolerant ones, due to a major availability of carbohydrates in these light‐rich habitats that can be destined to that reward (Schupp and Feener 1991).

Passifloraceae is the second family and *Passiflora* is the first genus with most records of EF nectary visiting ants, out of 65 families and 246 genera surveyed (Novais et al. 2025). Some attempts have explored the defensive function of the EF nectaries in *Passiflora* (Smiley 1985; Leal et al. 2006); a few others have probed that certain ants that visit the EF nectaries of *Passiflora* spp. reduce herbivore damage (McLain 1983; Smiley 1986). The ant guilds associated with co‐occurring *Passiflora* spp. vary between species with different nectary types and locations (Apple and Feener Jr 2001). The different recruitment of these hymenopterans to different *Passiflora* spp., either concerning density (Smiley 1986) or plant species involved (Lanza et al. 1993), seems to be related to the number of EF nectaries (McLain 1983; Smiley 1986) and to the amino acid composition and concentration in the respective passion vines. Other *Passiflora* spp. EF nectar utilization by ants has been mentioned (Ramírez et al. 2001; Wirth and Leal 2001; Oliveira Silva et al. 2016).

The existence of structures, either EF nectaries or protuberances in vegetative organs such as stipules, that simulate butterfly eggs have been reported for several *Passiflora* species (Gilbert 1982; MacDougal 2003). The presence of eggs, either real or not, diminishes butterfly oviposition in the surroundings (Williams and Gilbert 1981), and thus reduces herbivory (Mugrabi‐Oliveira and Moreira 1996).

The composition of the extrafloral nectar of *Passiflora* was studied in 18 species (Baker et al. 1978; Durkee 1983; Pascal 1993; Oliveira Silva et al. 2016; Monteiro Macedo et al. 2021; Ribeiro Soares et al. 2025). The amino acid complement of floral versus extrafloral nectar was compared by Baker et al. (1978). Durkee (1983) analyzed only the presence of different sugars and the concentration of sugars and amino acids, but not the individual compounds nor their quantities. Pascal (1993) analyzed the quantity and type of sugars, the total amount of amino acids, the presence of lipids, as well as the total percentage of the compounds and the volume secreted during the day. The presence of sugars was tested by Oliveira Silva et al. (2016, 2023) and Monteiro Macedo et al. (2021), who also checked for the presence of lipids, while Ribeiro Soares et al. (2025) compared the proportions of sugars.

However, considering this large genus, the constitution of the extrafloral nectar of many *Passiflora* species as well as the relation to their feeders remains unexplored.

The aims of this study were to describe the morphology of the EF nectaries and to compare the nectar secretion and composition of three species of *Passiflora* with different EF nectary types belonging to two subgenera: *P. caerulea* (subg. *Passiflora*), *P. suberosa* and *P. misera* (subg. *Decaloba*), as well as their relation (identity, occurrence, behavior) with nectar feeders and herbivores.

## 2 Methods and Materials

### 2.1 Species and Study Sites

*Passiflora* includes mainly tropical species, with a few subtropical ones (Feuillet and MacDougal 2007). *Passiflora caerulea, P. suberosa* and *P. misera* are lianas that occur from northern South America to Argentina (Killip 1938). In Argentina they are distributed from the north to the centre (Deginani 2001). The study plants of *P. suberosa* and *P. misera* were obtained from seeds collected in San Ignacio (Misiones province) and Concordia (Entre Ríos province), respectively; they were cultivated in a partially‐opened greenhouse of the Faculty of Exact and Natural Sciences of Buenos Aires University located in Ciudad Autónoma de Buenos Aires (C.A.B.A.). The greenhouse is a glass building of 100 m^2^, partially shaded by trees that grow around it, where plants from other species are cultivated and insect herbivores can access through the openings. The *Passiflora* plants were cultivated in pots (filled with black soil, sand and gravel 2:1:1) and watered daily, from 1998 to 2006. The pots were located in a 20 m^2^ sector of the greenhouse, intermingled arranged, where no plants of other genera were growing. Additionally, a specimen of *P. misera* cultivated in a garden in Merlo (Buenos Aires province), was studied; *P. caerulea* plants grew naturally in the mentioned garden. Nectar samples and the majority of observations on visitor activities on adult plants were taken in these localities at the end of summer, in the afternoon (between 2.30 and 7.30 p.m.), on February and March 2006; additional observations, about EF nectary visitors, concerning *P. suberosa* and *P. caerulea* were performed at Mburucuyá National Park (Corrientes province) during October 1998, as well as Merlo (Buenos Aires province) and Faculty of Exact and Natural Sciences of Buenos Aires University campus (C.A.B.A.), respectively, between 1991 and 2005, from 6 a.m. to 7.30 p.m. Weather in Argentina is characterized by an annual four stations temperature regime, being in average in Buenos Aires province and C.A.B.A. warm while in Corrientes province is hot. Number of plants sampled of *Passiflora caerulea, P. suberosa* and *P. misera* were 4, 8 and 3, respectively.

### 2.2 Nectary Morphology

The EF nectaries were observed at naked eye and with a hand lens in vivo in the field, as well as under a dissecting microscope in the laboratory both from fresh and dried herbarium leaves. The shape, color, location, and quantity were registered from 378 leaves from 15 plants. The nectary type was assigned following Elías (1983).

### 2.3 Nectar Sampling

Extrafloral nectar from nectaries of adult plants was collected during the day throughout 7 sunny days. Temperature and relative humidity were registered with a termohigrometer at each sampling occasion: they averaged 31°C and 59%, respectively. Nectar was only visible when ants were excluded. To exclude the ants, portions of stems were carefully shaken until no ant remained on them, then the EF nectaries were isolated by wrapping an aluminum paper strip around the base of the petioles of young leaves bearing them, and afterwards an ant deterrent resin was impregnated on the strip. Bridges between branches that could be used by ants to access the nectaries from other points were disentangled. Nectar was let to accumulate for at least 16 h, in order to get a measurable amount. Nectar was extracted with sterile calibrated end‐to‐end micro capillaries (Hirschmann Laborgeräte GmbH and Co. KG, Germany) and the volume registered. All the nectar that could be removed from a nectary was collected at each sample occasion from all the isolated nectaries in which it was available: *P. caerulea* (9–80), *P. suberosa* (7–98) and *P. misera* (4–18) nectaries. As the volumes were too little to assure the requested amount for the chemical screening, each sample consisted of nectar from more than one nectary of a single plant (the number of nectaries and the volumes that contributed to each sample were summed). In the case of *P. caerulea* (which has different nectary types in the same individual, described in the Results section), each sample consisted of nectar from the same nectary type (as referred to in the Results section), unless otherwise noted. During the whole manipulation of the samples, disposable nitrile gloves were worn to avoid contamination with the amino acids present on human hands.

### 2.4 Nectar Secretion

To estimate the secretion rate, the volume, number of nectaries from which the nectar was sampled, and hours of exclusion were recorded for each sample of the three species. To examine the effect of the removal on nectar production, sets of different nectary types of *P. caerulea* were sampled twice, at different time intervals.

### 2.5 Nectar Composition

Nectar composition was analyzed according to Piechowski (2007), as follows. Samples of nectar of the three species were fixed in Eppendorf vials previously filled with 1 mL of ethanol 70%. The nectar from the capillary was poured to the vial by means of a syringe applied to the capillary by a flexible plastic tube. Fixations were stored in a freezer until HPLC analysis at Ulm University.

The liquid content of the nectar samples had to be replaced by a known volume of water for the HPLC analyses. Therefore, the samples were vacuum‐centrifugated at 65°C (SpeedVac SC 110, Savant Instruments Inc., USA) until all liquidity vanished and only the nectar's solid components were left. These components were dissolved in 100 μL water (HPLC‐grade) and purified by filtering (0.45 μm pore size, Acrodisc syringe filter with nylon membrane, 4 mm, Pall Corporation, USA).

#### 2.5.1 Sugars

Following HPLC components were used: autosampler 717plus, pump 510, refractive index detector 410, and a column heater module (all Waters Corporation, USA). Isocratic separation was achieved using a 72:28 acetonitrile‐water mix (Schmidt 1998) with a flow rate of 1.4 mL min^−1^ and a high‐performance carbohydrate column (4.6 × 250 mm, Waters Corporation, USA) with a guard column. Operating temperature was 35°C. The mobile phase was degassed with helium 4.6 with a flow rate of 20 mL min^−1^. The system was monitored and data collected using the Millennium32 3.0.5 software (Waters Corporation, USA). External standards for sucrose, fructose, and glucose (Merck KGaA, Germany) were run. The HPLC output provided sugar concentrations in weight (μg) per sample volume (μL) for the single sugars. The concentrations had to be transformed into weight percent [sugar weight (μg) to total solution weight (μg) (w/w)] to provide comparability with published results; this unit is identical with the °Brix scale commonly used by refractometers (Bolten et al. 1979). Therefore, the sugar volume per sample volume (μL μL^−1^) had to be calculated by dividing the sugar weight by its density. The densities of the three occurring sugars are 1580.5 μg μL^−1^ for sucrose, 1600 μg μL^−1^ for fructose, and 1562 μg μL^−1^ for glucose (Lide 1995). Summing up the three sugar volumes resulted in the total sugar volume per sample volume. Subtracting this sugar volume from 1 resulted in the water volume (μL) per microliter of solution (with the simplified consideration of no further nectar components). The total solution weight (μg) per microliter of nectar was the multiplication of the water volume with 1000 and the addition of the total sugar weight (μg). Dividing the sugars weight by the total solution weight and multiplying the result with 100 resulted in the recommended weight percent unit.

The sugar ratio [sucrose/(fructose + glucose)] was calculated and assigned to the corresponding category defined by Baker and Baker (1983): sucrose dominant (> 0.9), sucrose rich (0.99–0.5), hexose rich (0.49–0.1), hexose dominant (< 0.1).

#### 2.5.2 Amino Acids

Amino acid analysis followed the AccQ•Tag method of the Millipore Corporation (1993). Samples (5 μL) were derivatized with AQC (6‐aminoquinolyl‐Nhydroxysuccinimidyl carbamate) in a borate buffer (without previous hydrolyzing). HPLC was performed using the following equipment: autosampler 717plus, 600E system controller, 470 scanning fluorescence detector, sat/in module, and a column heater module (all Waters Corporation, USA). Separation was achieved using an AccQ•Tag column (Novapak C18, 3.9 mm · 150 mm, Waters Corporation, USA) with a guard column. The gradient profile by Schmidt (1998) was used for the three eluents (a: sodium acetate, triethylamine (TEA) buffered with phosphoric acid (pH 5.5), ethylene‐diamine‐tetraacetic acid (EDTA); b: acetonitrile; c: water) with a flow rate of 1 mL min^−1^ at an operating temperature of 37°C. The mobile phase was degassed with helium 4.6 with a flow rate of 20 mL min^−1^. The system was monitored and data were collected using the Millennium32 3.0.5 software (Waters Corporation, USA). An external standard for alanine, arginine, asparagine, cysteine, glutamic acid, glycine, histidine, isoleucine, lysine, methionine, phenylalanine, proline, serine, threonine, tyrosine, valine, and leucine (amino acid hydrolysate standard, Waters Corporation, USA) was run.

### 2.6 Nectar Feeders and *Passiflora* Herbivores

The identity and the behavior of all the visitor taxa to the EFNs and herbivores of each *Passiflora* species were registered and observed during the day at the different locations. Records were performed by direct observation, *ad libitum*, through each day (Altmann 1984), the same days of nectar sampling, during 1 h each day, totaling 34 h of observation. One specimen of each taxon was captured for taxonomic determination. Ants were identified following Kusnezov (1978) and Fernández (2003a, 2003b).

### 2.7 Statistical Analysis

Sugar and amino acid variables were submitted to analysis using R (R Core Team 2025). Generalized linear models with a Gamma distribution and log link were used to assess differences in fructose, glucose, sucrose, and total sugar concentrations among *Passiflora* species, and also to compare extrafloral nectar secretion rates by hour and by individual nectaries among species. Additionally, amino acid composition and quantity were explored via a nonmetric multidimensional scaling (nmds) analysis with Bray–Curtis dissimilarity measure performed in the R environment with the vegan package functions (Oksanen et al. 2020). The dependent variables were nectar traits (volume, sugar quantity, amino acid quantity) while the independent variables were time of ant exclusion and species of *Passiflora*.

## 3 Results

### 3.1 Nectary Morphology

The EF nectaries of *P. caerulea* (Figure 1A) and *P. suberosa* (Figure 1B) are cup‐shaped and pedicelated, occur opposed in pairs along the petioles (petiolar), and additionally in the first species, in the stipule margins (stipular) and basal portion of the leaf sinuses (sinusal). There are 2 nectaries per petiole in *P. suberosa* whereas there are 2–5 per petiole, 2–5 per stipule and 5–12 on the sinuses in *P. caerulea* , the modes being 2, 3 and 8, respectively. The nectaries of *P. caerulea* are brownish and the ones of *P. suberosa* are green. The EF nectaries of *P. misera* (Figure 1C) are ocellate, sessile, without protruding beyond the leaf blade surface, yellow, visible from the adaxial leaf face but the secretion is accessible from the abaxial one; there are generally 2 at the leaf blade base (rarely 0, 1 or 3), each one at each side of the central vein, the rest (2–6) distribute more or less symmetrically between the central vein and each of the lateral ones (aligned along the bisectings of the central and each of the remnant 2 main veins but not equidistant from the ones of the same line). Teratological nectaries were observed in one specimen of *P. suberosa* ; they were ocellar and sparse in the leaf blade, as the ones of *P. misera* (Figure 1B, insert).

**FIGURE 1:**
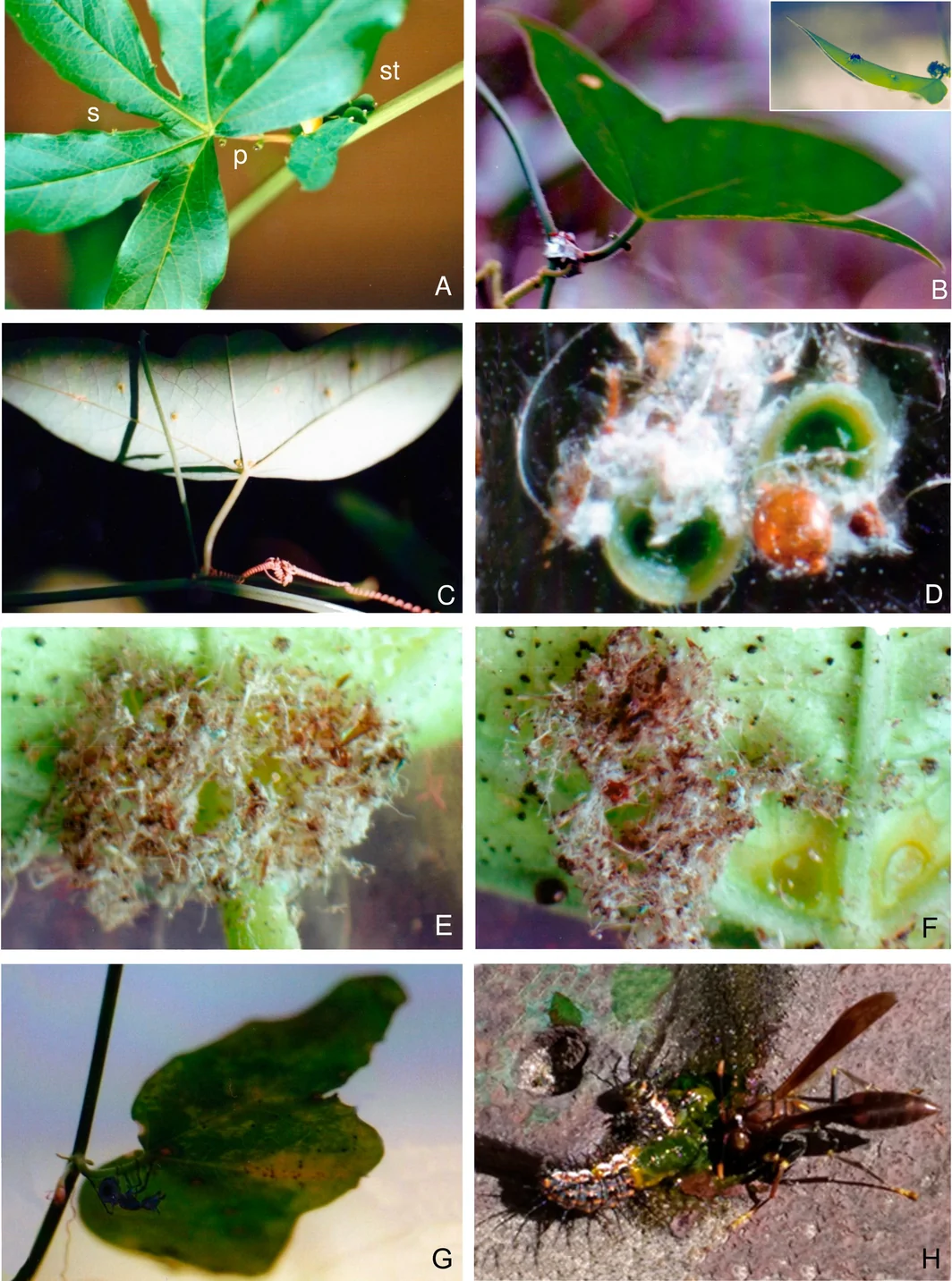
Extrafloral nectaries of *Passiflora* spp. and related ant and wasp activity. (A) Cup‐shaped nectaries of *P. caerulea* (p, petiolar; s, sinusal; st, stipular). (B) Cup‐shaped petiolar nectaries of *P. suberosa* (insert: teratological ocellar laminar nectaries of *P. suberosa* and *Camponotus mus* ant licking one of them). (C) Ocellar laminar nectaries of *P. misera* . (D) Non‐longer secreting extrafloral nectaries of *P. suberosa* detached from a leaf with detritus removed from their interior. (E) Debris covering basal extrafloral nectaries of *P. misera* gathered by *C. iheringii* ants. (F) The same as F but debris put aside to show the two basal ocellated extrafloral nectaries. (G) *Camponotus mus* ant in defensive position on a *P. misera* leaf. (H) *Polistes* sp. preying on a *Dione vanillae maculosa* caterpillar removed by the wasp from a *P. caerulea* plant.

### 3.2 Nectar Secretion

In the three species, although nectaries form in early stages of development, saplings (plants with protophylls) of the *Passiflora* species growing simultaneously with adults (plants with nomophylls) did not produce extrafloral nectar nor were patrolled by ants. Within each adult plant, only young leaves secreted nectar; nectaries of older, coarser, usually larger leaves became dry in all the species; the ones of *P. suberosa* , the larger ones, were filled with detritus and Acari were found inside (Figure 1D). In *P. caerulea* , nectaries of the same leaf but of different type were functional simultaneously first, but the sinusal nectaries continued secreting while the petiolar ones were no longer functional.

The secretion rate did not differ significantly between *P. misera* and *P. caerulea* (1.75‐fold higher in *P. misera* , *p* = 0.250), but the one of *P. suberosa* was higher than the one of *P. caerulea* (2.62‐fold higher, *p* = 0.031) (Figure 2). The increase in the time after ant exclusion had no effect in nectar production in any of the species: *p* = 0.3904, 0.3911 and 0.9321, for *P. caerulea, P. misera* and *P. suberosa* , respectively (Figure 3); in *P. caerulea* , neither when all the nectaries of different type were plotted together (Figure 3) nor when petiolar nectaries were isolated analyzed (0.04 μL during 16 h exclusion versus 0.002 μL during 48 h exclusion). Nevertheless, when a second extraction was performed to nectaries of *P. caerulea* , they had secreted again and even faster, being the difference, for the petiolar ones, more than the double after 1/3 of the previous exclusion time lapse (Table 1).

**FIGURE 2:**
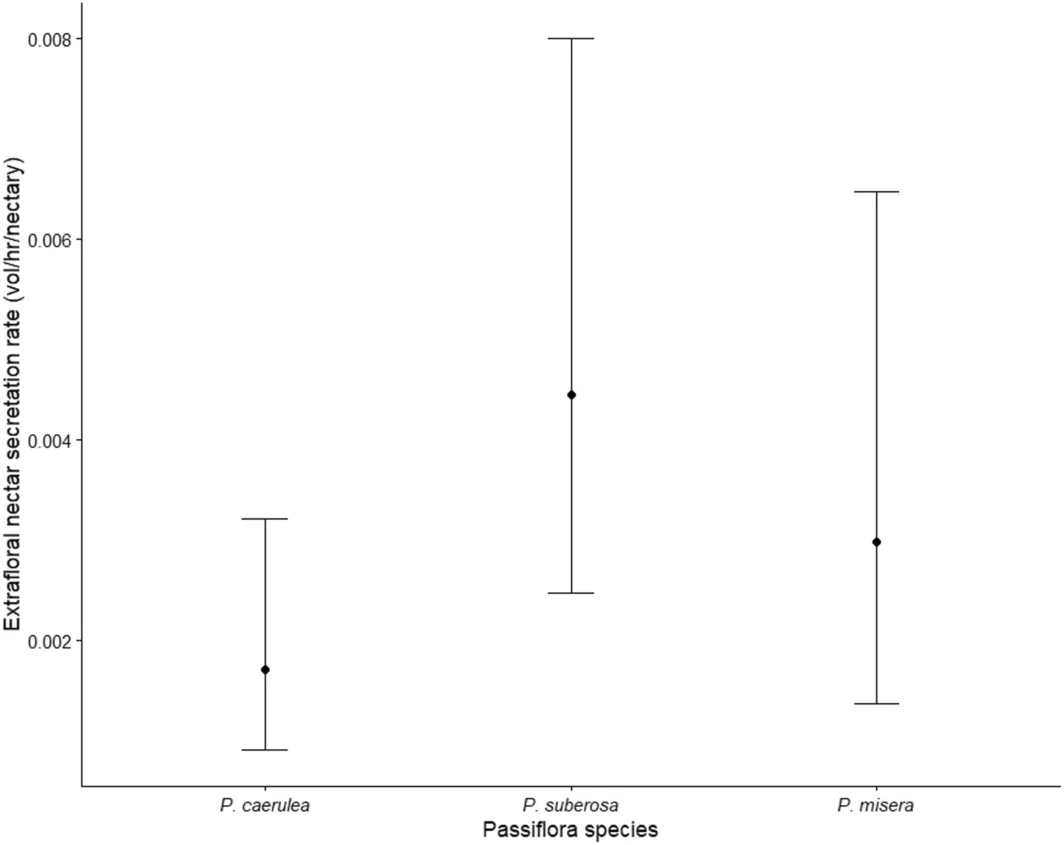
Extrafloral nectar secretion rate (vol/h/nectary), in microliter, of the three *Passiflora* species studied.

**FIGURE 3:**
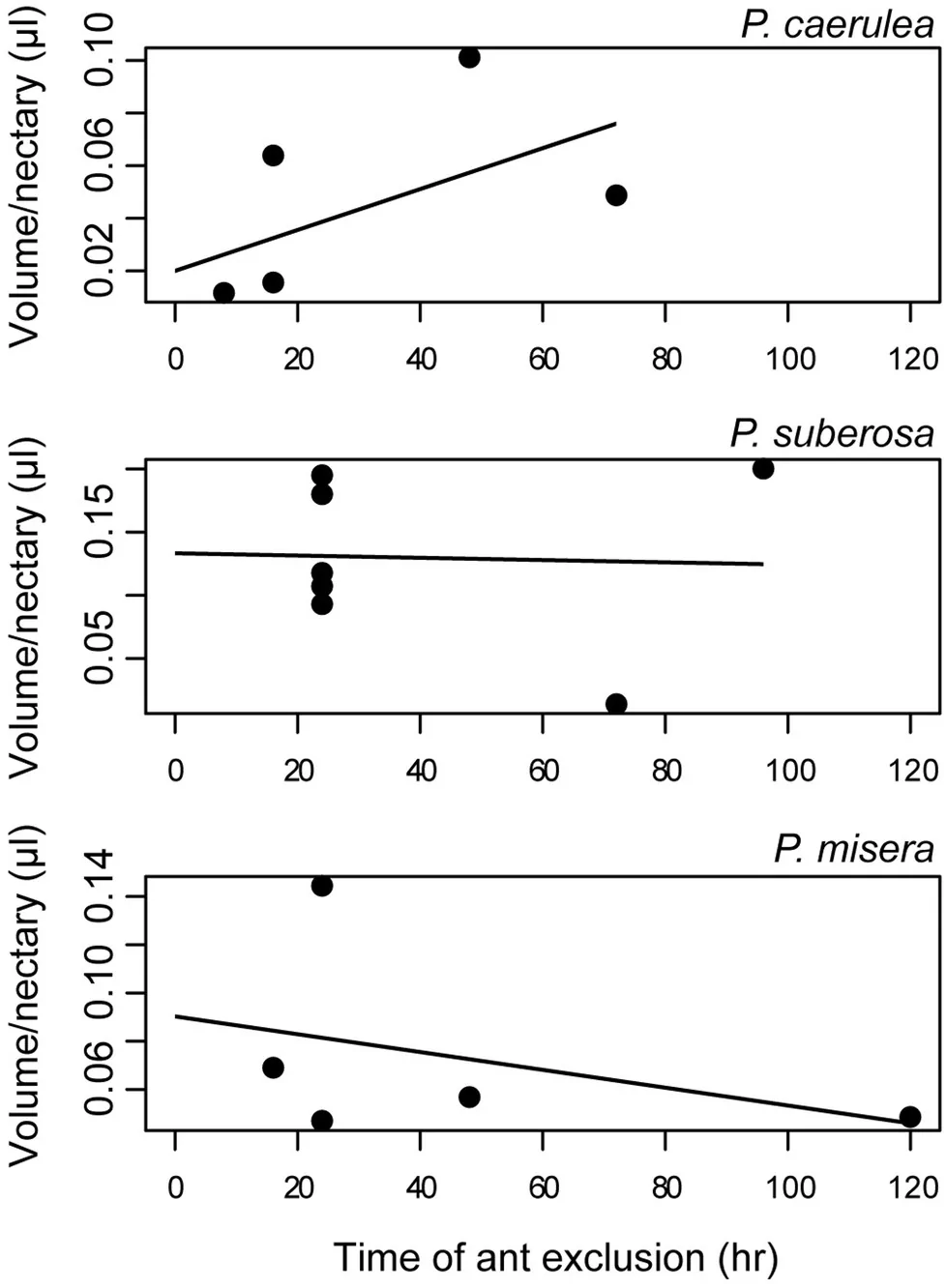
Extrafloral nectar secretion of *Passiflora* spp. as a function of the time after ant exclusion. Each point represents a sample of nectar from nectaries that had been submitted to the corresponding time of ant exclusion in the *x* axis. Regressions were not significant for any species.

**TABLE 1:** Effect of removal on extrafloral nectar production in *P. caerulea* extrafloral nectaries.

Sample code/Nectary type (quantity of sampled nectaries)	119/Stipular (80)	124/Petiolar (10)	126/Petiolar (10)
Volume of 1st extraction	0.56	0.12	0.33
Time (h) of ant exclusion until 1st extraction	16	72	72
Volume of 2nd extraction	0.7	0.31	0.70
Time (h) of ant exclusion until 2nd extraction	24	24	24

*Note:* Units are in microliter.

### 3.3 Nectar Composition

Fructose, glucose and sucrose were present in 33 out of 35 samples of every nectary type of the three species (Table 2), except for only one of *P. suberosa* , in which glucose was absent; 2 samples, one of *P. suberosa* and one of *P. caerulea* exhibited no sugars. The total amount of sugars did not differ significantly among species (Figure 4), with neither *P. suberosa* nor *P. misera* differing from *P. caerulea* (*p* = 0.448 and *p* = 0.313, respectively). Considering individual sugars, no differences were detected among species in fructose or glucose amounts: fructose amounts in *P. suberosa* and *P. misera* were 0.76 and 0.96 times those of *P. caerulea* , respectively (*p* = 0.107 and *p* = 0.797), while glucose amounts were 0.80 and 1.01 times those of *P. caerulea* (*p* = 0.227 and *p* = 0.976). In contrast, sucrose amounts were 55.8% higher in *P. suberosa* than in *P. caerulea* (*p* = 0.005) and 28.5% lower in *P. misera* than in *P. caerulea* (*p* = 0.030). The sugar ratio [sucrose/(fructose + glucose)] was sucrose rich for *P. caerulea* and *P. misera* , while it was sucrose dominant for *P. suberosa* . In *P. caerulea* , when the sugar properties of the different nectary types were analyzed separately, no significant differences arose in the amount of total sugars nor in the individual sugars (Wilcoxon *p*‐rank values: total = 0.28; fru = 0.28; glu = 0.28, suc = 0.78) but in the sugar ratio: stipular and sinusal nectars were sucrose rich while petiolar nectar tended to be sucrose dominant (Table 3).

**TABLE 2:** Total amount of sugars, quantity of each sugar type and sugar ratio in the extrafloral nectar of *Passiflora* species.

Species	*P. caerulea*	*P. suberosa*	*P. misera*
Total sugar amount	54.12 ± 20.85	60.72 ± 18.45	46.42 ± 16.82
Fructose	15.05 ± 6.31	11.49 ± 3.59	14.43 ± 5.84
Glucose	13.92 ± 6.18	10.06 ± 4.98	13.99 ± 5.92
Sucrose	25.15 ± 9.79	39.17 ± 2.11	18.00 ± 5.89
Sugar ratio (sucrose/fructose +glucose)	0.94 ± 0.27	1.88 ± 0.55	0.68 ± 0.19
Sucrose dominance	sr	sd	sr
Number of samples (plants)	13 (4)	10 (7)	10 (3)

*Note:* All values are means ± standard deviation measured in weight/weight (%).

Abbreviations: sd, sucrose dominant; sr, sucrose rich.

**FIGURE 4:**
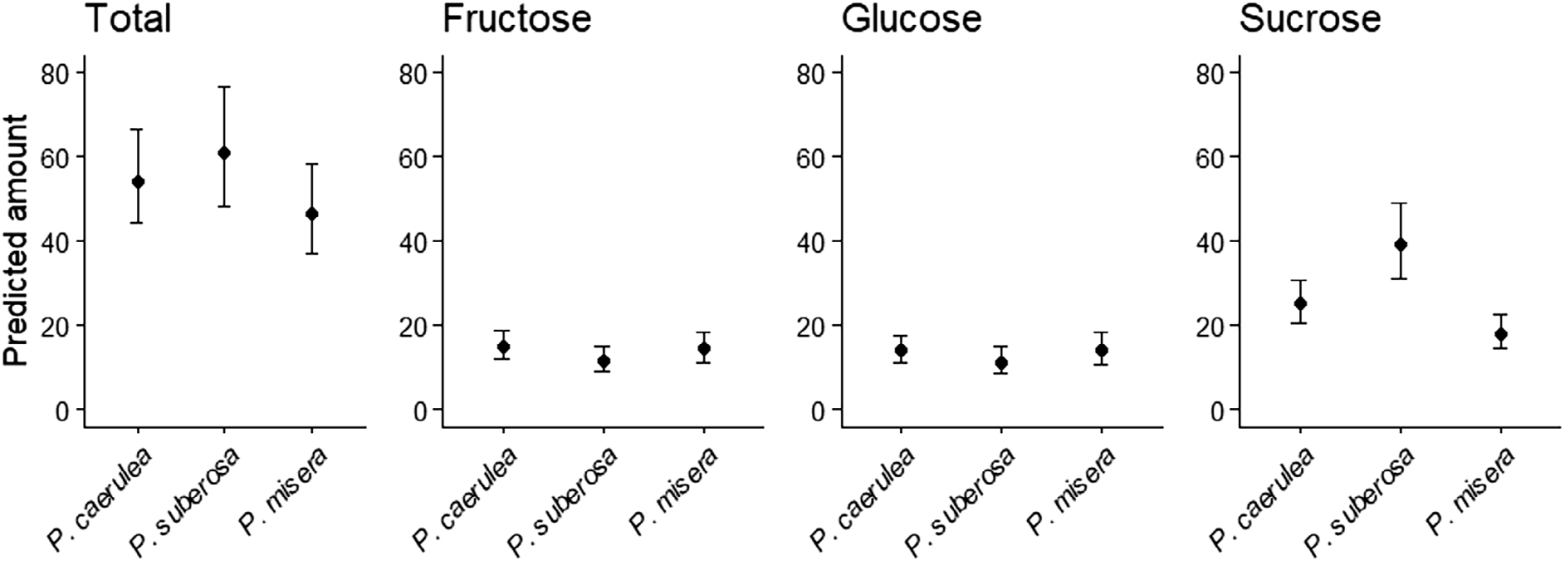
Predicted sugar amount (total and of each sugar) measured in weight/weight (%) of the three *Passiflora* species studied.

**TABLE 3:** Total amount of sugars, quantity of individual sugars and sugar ratio in the different nectary types of *Passiflora caerulea* .

Nectary type	Petiolar	Sinusal	Stipular
Fructose	12.36 ± 5.60	18.70 ± 6.85	22.6
Glucose	11.15 ± 5.30	17.99 ± 6.91	20.53
Sucrose	23.27 ± 11.66	28.69 ± 6.52	23.07
Total	46.77 ± 21.89	65.38 ± 19.17	66.2
Sugar ratio	1.06 ± 0.27	0.84 ± 0.23	0.54
Sugar dominance	Sd	sr	Sr
Samples (*n*)	8	3	1

*Note:* All values are means ± standard deviation measured in weight/weight (%).

Abbreviations: SD, sucrose dominant; sr = sucrose rich.

The amino acid total amount (Table 4) was threefold lesser in *P. caerulea* than in *P. suberosa* and *P. misera* (*F* = 8.31, *p* < 0.05). The number of amino acids of *P. misera* was larger than the one of *P. suberosa* , which was larger than the one of *P. caerulea* (Table 4), and was quite variable for each species (Table 5, see range). Of the 17 proteinogenic amino acids analyzed, 15 were found, but not together in any sample (Table 5); cysteine and methionine were absent from all samples (Table 5); arginine, threonine, alanine, proline and lysine were the most frequent (Table 5); asparragine and glycine appeared in only one sample (but not the same) of *P. misera* . The presence as well as the quantity of each amino acid varied between the species and within every species (Table 5, see ranges); though, analyzing these traits together via nmds (which reached a stress value of 0.0775 after 20 runs), every species was rather clearly defined (Figure 5): only *P. suberosa* and *P. caerulea* overlapped, but very little; *P. misera* was more represented by high levels of, for example, leucine, isoleucine, valine, serine while *P. suberosa* by alanine, arginine, proline, and *P. caerulea* by threonine and lisine; samples of *P. misera* were the least variable while the ones of *P. caerulea* were the most (due to two particularly different samples). Comparing the amino acids profiles of the different nectary types of *P. caerulea* , the total amount was quite similar; the only apparent difference was the lysine content (usually nearly the half in the petiolar nectaries as compared to the stipular and sinusal ones) (Table 5).

**TABLE 4:** Amino acid content of extrafloral nectar of *Passiflora* species.

Species	*P. caerulea*	*P. suberosa*	*P. misera*
Total amount (ng/μL) (mean ± SD)	3970.83 ± 1938.67^a^	11 944.04 ± 5826.97^b^	11 561.40 ± 6364.45^b^
Total quantity of amino acids (range)	2–9	5–13	11–14
Number of samples (plants)	9 (3)	8 (6)	7 (3)

*Note:* Different superscript letters indicate significant differences between the species.

**TABLE 5:** Amino acid profiles of extrafloral nectar of *Passiflora* species (ng/μL).

	S	asp	ser	lu	glu	his	arg	thr	ala	pro	cys	tyr	val	met	lys	ile	leu	phe	Total
*P. caerulea*	116p	0	229.50	0	0	759.90	464.89	358.29	173.18	1840.00	0	0	227.69	0	287.23	265.51	0	0	4606.18
	123 s	0	0	0	0	1027.79	1247.15	924.33	637.89	1783.03	0	0	0	0	0	0	0	0	5620.20
	130st	0	330.03	0	0	0	0	0	0	0	0	0	0	0	366.72	0	0	0	696.74
	131p,s	0	191.83	0	0	331.10	438.05	320.40	277.93	787.20	0	326.75	0	0	189.02	0	0	0	2862.28
	135p	0	0	0	0	0	428.13	293.73	191.97	350.49	0	0	0	0	195.52	0	0	0	1459.85
	118p	0	0	0	0	0	1642.23	1206.28	964.47	1682.31	0	0	0	0	1163.39	0	0	0	6658.67
	132p	0	0	0	0	0	891.35	630.23	452.34	1011.63	0	724.11	0	0	506.42	0	0	0	4216.09
	119 s	0	380.66	0	0	762.87	555.66	498.48	447.03	1759.82	0	0	0	0	426.62	0	0	0	4831.12
	120p	0	245.38	0	0	706.99	668.52	407.12	209.86	1788.43	0	0	261.54	0	189.72	308.77	0	0	4786.32
	10p	0	0	0	0	0	58.45	0	34.89	341.22	0	0	0	0	0	0	0	0	434.56
	Rg	0	0–380.66	0	0	0–1027.79	0–1642.23	0–1206.28	0–964.47	0–1840.00	0	0–724.11	0–261.54	0	0–1163.39	0–308.77	0	0	
*P. suberosa*	99	0	0	699.29	0	958.09	2871.71	1253.11	942.58	2146.42	0	0	0	0	849.19	0	0	0	9720.40
	100	0	0	0	0	0	1115.72	807.95	416.00	1314.99	0	0	0	0	625.55	0	0	0	4280.20
	103	0	923.34	0	0	1220.55	1750.35	902.44	475.88	4034.62	0	810.46	481.16	0	396.77	0	791.61	1009.46	12 796.64
	104	0	806.51	0	0	1244.54	1782.94	1070.49	639.09	1536.13	0	1372.88	0	0	766.54	0	1118.22	1352.07	11 689.42
	106	0	1097.82	707.49	0	1439.92	2312.52	991.53	691.59	4141.29	0	888.73	620.01	0	634.95	709.11	1089.37	1522.83	16 847.14
	114	0	462.95	0	0	715.65	1051.46	639.46	535.21	1037.67	0	718.62	443.71	0	423.44	491.59	636.01	871.37	8027.14
	115	0	647.44	0	0	943.19	1434.76	795.38	498.16	2143.84	0	0	480.17	0	489.03	0	693.92	921.71	9047.67
	Rg	0	0–1097.82	0–707.49	0	0–1439.92	1051.46–18 440.69	639.46–1253.11	416.00–942.58	1037.67–4141.29	0	0–1372.88	0–620.01	0	396.77–849.19	0–709.11	0–1118.22	0–1522.83	
*P. misera*	98	692.94	2642.71	832.91	0	2690.25	1360.87	869.67	1785.62	3398.28	0	1813.11	678.38	0	594.81	706.07	2507.17	2808.00	23 380.78
	105	0	811.49	464.44	0	655.43	609.90	242.16	670.37	1019.41	0	657.00	263.32	0	263.58	286.87	960.06	1166.46	5393.53
	111	0	698.80	392.27	0	547.82	636.93	352.51	286.18	1429.57	0	617.66	300.45	0	270.02	337.95	603.46	825.23	7298.85
	112	0	1526.65	295.10	144.29	767.01	807.18	408.15	1801.17	2947.81	0	990.69	346.76	0	183.47	379.31	1232.97	1712.23	13 542.79
	113	0	1183.47	694.58	0	1041.71	1185.00	785.00	1225.41	2507.72	0	1296.56	522.12	0	467.74	586.92	1511.82	2092.34	15 100.38
	125	0	755.10	0	0	1006.33	1002.55	637.77	634.88	891.96	0	990.32	470.47	0	443.06	0	964.41	1271.84	9068.68
	134	139.82	515.11	176.06	0	508.64	308.44	194.78	270.02	512.67	0	307.89	112.87	0	121.62	134.81	530.32	634.60	4467.64
	Rg	0–692.94	515.11–2642.71	0–832.91	0–144.29	508.64–2690.25	308.44–1360.87	194.78–869.67	270.02–1801.17	512.67–3398.28	0	307.89–1813.11	112.87–678.38	0	121.62–594.81	0–706.07	530.32–2507.17	634.60–2808.00	

Abbreviations: p, petiolar nectaries; Rg, range; S, sample; s, sinusal nectaries; st, stipular nectaries.

**FIGURE 5:**
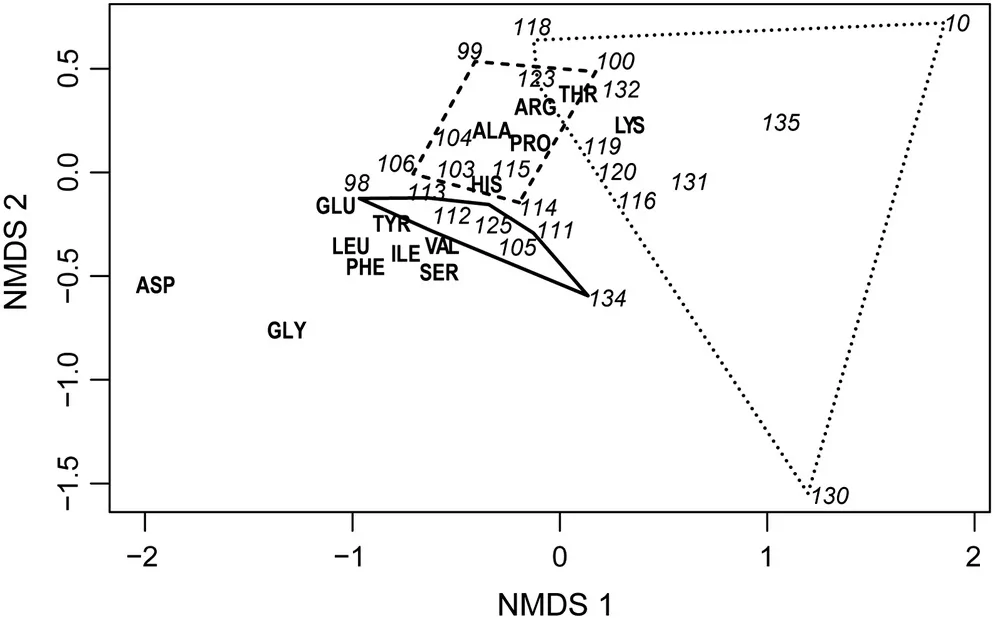
Amino acid complement of *Passiflora* spp. Polygones include samples (numbers, cf. Table 5) of each species: Full line, *P. misera*; dashed line, *P. suberosa*; dotted line, *P. caerulea*. Position of labels was slightly jittered to improve the figure clarity.

### 3.4 Nectar Feeders and *Passiflora* Herbivores

Co‐occurring plants of different species shared visits to EF nectaries of some insect taxa but the visitor assembly on a single plant species varied between sites (Table 6). Workers of the ants *Camponotus mus* licked the teratological nectaries of *P. suberosa* that resembled the ones of *P. misera* (Figure 1B, insert), but not the common and more abundant petiolar ones, which were only visited by the ant *Crematogaster iheringii*. On *P. misera* , a barrier of debris (mostly molts and feces of Tingidae bugs) was found neatly gathered around the perimeter of some nectaries while *C. iheringii* ants licked inside (Figure 1E,F). When the barrier was disintegrated and observed with a dissecting microscope, living Acari appeared among the detritus. At P. N. Mburucuyá, ants did not patrol *P. suberosa* before 9 a.m., though nectar drops accumulated on the nectaries since 8 a.m. in spite no exclusions were performed; afterwards, the nectar drops began to reduce. *Camponotus mus* individuals visited more than one nectary of the same leaf when patrolling; sometimes, they even licked again the same nectary that they had recently licked. When different ants encountered on their opposite ways along the stems, they stopped for a second while their antennae got in touch and each of them continued on the previous direction of their ways afterwards. The wasp *Polybia occidentalis scutellaris* from a nest hanging from a tree near a *P. misera* specimen in Merlo visited the EF nectaries of this passion vine. The phytophages *Dione vanillae maculosa* and *Holymenia hystrio* were omnipresent in all localities and *Passiflora* spp., while the rest were present on some species and localities, or either in the wild or in the greenhouse (Table 7). *Dione vanillae maculosa* butterflies oviposited several eggs during each laying bout, but one at each site (on the passion vines leaves or tendrils or on other plant species or nonliving substrates adjacent to passion vines) and the caterpillars ate the leaves. The adults of *Cacoscelis melanoptera* fed on the leaves and flowers while the adults of *Systena* sp. fed on the leaves. Nymphs and adults of *Holymenia histrio* sucked sap from blossoms, flowers and fruits, either ripe or not. Nymphs and adults of *Gargaphia lunulata* sucked sap from leaves; the eggs were laid inside the leaf veins. All the known instars of the mentioned taxa spent their life stages on the passion vines, where they fed and copulated, except from the butterfly.

**TABLE 6:** Arthropod presence and activity at extrafloral nectaries of *Passiflora* spp. in the different study sites.

*Passiflora* sp.	*P. caerulea*		*P. suberosa*		*P. misera*	
Taxa	Merlo	C.A.B.A.	P. N. Mburucuyá	C.A.B.A.	Merlo	C.A.B.A.
**Hymenoptera**						
Formicidae						
SubF.Formicinae		+		+ (l)	+	+
*Camponotus mus*						
SubF.Myrmicinae	+					
*Crematogaster iheringii*	(st, s, p)					
Vespidae				+	+	+
Unidentified	+		+		+	
*Polistes* sp.	−			−	+	−
*Polybia occidentalis scutellaris*					+	
Apidae (afin.)					+	
**Diptera**						
Muscidae					+	
Lonchaeidae						
sp. 1	+ (p)				+	
sp. 2					+	
**Hemiptera**						
Tingidae						
SubF. Tinginae						
*Gargaphia lunulata*						+
Berytidae						
SubF. Metacanthinae				+		

Abbreviations: −, not licking; +, licking; l, laminar; p, petiolar; s, sinusal; st, stipular.

**TABLE 7:** Phytophagous insects of *Passiflora* species at the different localities.

*Passiflora* sp.	*P. caerulea*		*P. suberosa*		*P. misera*	
Taxa	Merlo	C.A.B.A	P. N. Mburucuyá	C.A.B.A	Merlo	C.A.B.A
Lepidoptera						
Nymphalidae						
Heliconiinae						
*Dione vanillae maculosa*	+	+	−	+	+	+
Coleoptera						
Chrysomelidae						
Halticinae						
*Cacoscelis melanoptera*	+	+	−	−	−	−
*Systena* sp.	+	+	−	+	−	+
Hemiptera						
Coreidae						
*Holymenia histrio*	+	+	−	+	+	+
Tingidae						
Tinginae						
*Gargaphia lunulata*	−	+	−	−	−	+

Abbreviations: −, absence; +, presence.

Interactions of visitors to the EF nectaries with phytophages were as follows: *Camponotus mus* chased away larvae of *H. hystrio* and of *D. vanillae maculosa*. This deterrence was temporally brief, more annoyance than dissuasion: that is, nymphs of *H. histrio* walking along the vines stems turned and walked in the opposite direction when they encountered an ant, but they did not abandon the plant. Something similar occurred with the lepidopteran larvae: when *C. mus* ants, in their way between EF nectaries, met one caterpillar, seemed to bite it; after this, the caterpillars stopped chewing but they remained in their location, or, at least, moved the anterior part of the body to a side; they could stay ca. 10 min in that position, but when the ants went away, they continued feeding. Some *C. mus* ants remained immobile on *P. misera* , with the abdomen pointed forwards and the mandibles open, the position to spray their formic acid (Figure 1G). On *P. caerulea*, they walked on the leaf blades and along the stems with the abdomen in such position when there were recently emerged caterpillars. The wasp *Polistes* sp. preyed on *D. vanillae maculosa* caterpillars, throwing them to the ground, biting them and flying with a portion of the body between its mandibules (Figure 1H).

## 4 Discussion

The goals of this research were to study the EF nectary morphology and nectar composition of three *Passiflora* species with different nectary types included in two subgenera, as well as their association with nectar feeders and herbivores. For that, the sugar and amino acid profiles together with nectar secretion were compared, and the identity, occurrence and activity of nectarivores and phytophages was surveyed in different sites. Nectar was produced only in young leaves, independently of ant exclusion time but again and faster after experimental removal. No longer functional nectaries of *P. suberosa* as well as the detritus buildings of *C. iheringii* around the nectaries of *P. misera* served as incidental domatia housing Acari. *P. caerulea* and *P. misera* EF nectaries, due to their color, shape and distribution, would mimic lepidopteran eggs. The total sugar amount and the high sucrose/hexose ratio were similar between species, but the amino acid quantity and quality of *P. caerulea* differed from those of *P. suberosa* and *P. misera* (which belong to a different subgenus), thus amino acid traits might contribute to taxonomy. Co‐occurring specimens of different plant species shared only some nectarivores, and not all insect taxa were associated with each plant species in every site. Ants and wasps seemed to protect *Passiflora* spp. from Heliconiinae caterpillars and Coreids; other hemipterans and flies stole nectar. Following, each main treated theme will be discussed.

### 4.1 Nectary Morphology

The cup shaped and pedicelated nectaries of *P. caerulea* and *P. suberosa* versus the ocellate and sessile ones of *P. misera* belong to the elevated and flattened types, respectively, in the classification of EF nectaries made by Zimmerman (1932 cited in Elías 1983). Teratological nectaries had been mentioned for *P. suberosa* (Cusset 1965), but concerning location, not nectary type. Interestingly, only *C. mus* , a permanent visitor of *P. caerulea and P. misera*, visited the teratological nectaries of *P. suberosa* , which resembled the ones of *P. misera* , in the site where *P. suberosa* was growing in sympatry with *P. misera* , while *C. iheringii* limited its visits to the normal nectaries of *P. suberosa* . This may be related to the visual recognition of an already exploited food source, as vision takes part in the orientation of most ants (Klaus Jaffé et al. 1993), which besides is expected in located‐predictable food sources as EF nectaries (Caroll and Janzen 1973).

EF nectaries of *P. misera* and *P. caerulea* are probably Heliconiinae egg mimics. Petiolar EF nectaries of *P. caerulea* were suggested to mimic eggs (Sacchi 1988 cited in Jolivet 1992). The size of these nectaries and that of the eggs of *D. vanillae maculosa* is similar. Eggs of *A. vanillae maculosa* are ovate: when laid, their outline appears oval in lateral view but rounded seen from above, so they could be mistaken with either the ocellate EF nectaries of *P. misera* or the cup‐shaped ones of *P. caerulea* seen from above. Considering other passion vines, Gilbert (1975) suggested that the color of the fake eggs resembles the color of the eggs close to hatch rather than the color of newly oviposited ones. Recently laid eggs of *D. vanillae maculosa* are yellow, then they turn grayish‐brown; a similar color change was reported by Ajmat de Toledo (1991). So EF nectaries of *P. misera* would resemble recently oviposited eggs while those of *P. caerulea* would resemble eggs ready to hatch. Some passion vine butterflies lay the eggs isolated while others lay them in clusters (Boender 2004). The last ones are not known to oviposit on *Passiflora* species lacking egg mimics (Benson et al. 1975). The isolated condition of the eggs of *D. vanillae maculosa* supports the idea that EF nectaries of *P. misera* might mimic these butterfly eggs. In some *Passiflora* species, the fake eggs on the leaf blades are randomly distributed (Gilbert 1982); in *P. misera* , they distribute more symmetrically, but the lack of equidistance may reinforce the trick.

### 4.2 Nectar Secretion

The active EF nectaries in the more vulnerable younger leaves of the three species studied coincide with observations in other *Passiflora* species (MacDougal 1994) and most explored genera (Elías 1983; Bentley 1977; Majer 1979; Keeler 1980; Koptur 1984, 1989; Vilela and Palacios 1997; Heil et al. 2000). In contrast, EF nectaries of *Ipomoea carnea* are functional in young and mature leaves (Koptur 1989) and the ones of *Leonardoxa africana* (Fabaceae) begin to secrete when leaves are mature (Gaume and McKey 1999); *L. africana* also provides shelter inside domatia to ants (Gaume and McKey 1998), so the patrolling, even of non‐secreting leaves, is assured by another and permanent reward. The ant presence in young leaves of adult plants is in accordance with EF activity in the three species studied. Accordingly, in *Passiflora mooreana*, ants tend to patrol whole young plants (when plants have not surpassed 1.5 m high) and young leaves of adult plants, but they are absent from plants without sprouts and coarser leaves (M. T. Amela García, unpublished data).

The resumption, even an increase in secretion rate, after nectar removal in *P. caerulea* and the lack of relation of nectar volume with time in the three studied species suggest that EF secretion is stimulated by consumption and could constitute an inducible defense. This effect was also detected in *Macaranga tanarius* (Heil et al. 2000) and was demonstrated with simulated herbivory in *Vicia sativa* (Koptur 1989) and in *M. tanarius* (Heil 2008) as increased nectar production, in *Impatiens sultani* as major amino acid content (Lanza et al. 1993), in *Vicia faba* as additional nectaries formation (Mondor et al. 2006) and in *Qualea multiflora* as higher volume and calories (Calixto et al. 2020). This would attract more ants, as their abundance is positively correlated with increasing nectar production (Bentley 1983; Dreisig 2000), when the plant needs protection, instead of assigning energy to a more expensive constitutive defense (Bixenmann et al. 2011).

The presence of Acari in nectaries no longer producing nectar of *P. suberosa* as well as among the detritus gathered by *C. iheringii* around the basal nectaries of *P. misera* suggests that these glands and surrounding ant buildings might function as non‐true crateriform domatia used by opportunistic Acari. Non‐true domatia are varied plant structures that give refuge to ants (Delabie et al. 2003) or to other arthropods, such as Acari (O'Dowd and Willson 1989), that are beneficial for the host plant. The detritus accumulated inside the nectaries of *P. suberosa* or the ones gathered by the ants remind one of the hair tufts common in some mite domatia (Jolivet 1998; Heil 2008). Predatory mites associated with EF nectaries which presumably protect plants against herbivorous mites have been reported (Walter and O'Dowd 1995).

### 4.3 Nectar Composition

Sugar concentration of the EF nectar of the three studied passion‐vines was about 50%, the common value for the exposed EF nectar in sunny or dry climate (Koptur 1992); anyway, in *P. misera* , whose nectaries are more protected from evaporation (on the abaxial leaf surface), nectar was more diluted. EF nectar sugar concentration ranges between 27%–53% in other *Passiflora* spp. (Durkee 1983), 28%–47% in *Inga* spp. (Koptur 1984), 50%–60% in *Vigna unguiculata* (Pate et al. 1985), and 8%–40% in members of several families according to nectary type (Díaz‐Castelazo et al. 2005); these last authors recorded lower (though not significantly) sugar concentration in flattened nectaries than in capitate or peltate ones, which is in accordance with the lower concentration of the flattened nectaries of *P. misera* as compared with the pedicelated cup shaped ones of the other two species.

The three species contained fructose, glucose, and sucrose, typical carbohydrates of EF nectar (Koptur 1992), as exemplified in two species of *Vicia* (Percival 1961), six species of *Passiflora* (Durkee 1983; Pascal 1993), *Norantea brasiliensis* (Gottsberger et al. 1984), *Vigna unguiculata* (Pate et al. 1985), and the majority of the samples of 16 species of different families (Blüthgen et al. 2004); less frequently, EF nectar may contain only glucose and fructose (Keeler 1980), sucrose and glucose, or only sucrose (Galetto and Bernardello 1992). Concentration of these individual sugars in the studied *Passiflora* species fit the most common values found in the taxa examined by Blüthgen et al. (2004).

The species analyzed in this study had sucrose‐rich or sucrose‐dominant nectar. These sugar ratios occur in most EF nectars (Koptur 1992; Pascal 1993) but not in all (Gottsberger et al. 1984). The relative proportions of sugars encountered in *P. caerulea* disagree with the estimations of Durkee (1983) for this species, probably made by means of paper chromatography (a less accurate technique).

The amino acids encountered in this study are the usual ones in EF nectars of other taxa (Keeler 1977; Baker et al. 1978; Inouye and Inouye 1980), as well as their frequency (Baker et al. 1978), even though the number and the combinations varied between taxa. The number of amino acids fell in the ranges found for EF nectars of other species (Baker et al. 1978; Heil et al. 2000). Some amino acids appeared in most of the samples while others in fewer ones; such variability between samples had been reported in other genera (Inouye and Inouye 1980; Blüthgen et al. 2004; Rudgers and Gardener 2004). Of the 10 amino acids required by insects (Koptur 1992), arginine, histidine, isoleucine, lysine, threonine, tyrosine, valine were present in at least some of the samples of the three species; surprisingly, the sulfur‐containing amino acids, cysteine and methionine, quite common in EF nectars (Baker et al. 1978) did not appear in any sample, whereas leucine and phenylalanine were absent in *P. caerulea* . Of the “quasi essential” and “common” amino acids (Gottsberger et al. 1984), alanine and serine were present in the three species, but glycine and asparagine did neither occur in *P. suberosa* nor in *P. caerulea* , and glutamic acid was absent in *P. caerulea* . Proline was present and it was the most abundant amino acid in almost all the samples of the three species. Abundance of this amino acid was also reported for non‐floral nectar sources (Blüthgen et al. 2004).

The EF nectar of the different EF nectaries of *P. caerulea* varied little (in sugar ratio and in lysine content). No significant differences were encountered between nectaries located in vegetative parts versus EF nectaries associated to inflorescences in the occurrence of individual amino acids of 21 and 12 species, respectively (Baker et al. 1978), nor in carbohydrate or total amino acid concentration in cotton (Rudgers and Gardener 2004); only slight differences in nectar composition between nectary types were found in *Campis* x *tagliabuana* (Baker et al. 1978); in *Vigna unguiculata* , the ones of the inflorescences have higher amino acid levels than the ones of the leaves but the proportion of sugars was similar (Pate et al. 1985). In spite of being associated to distinct plant structures, as the function is the same (attraction to ants which in turn would repel plant damaging insects), no great differences would be expected about nectar composition in relation to nectary location.

The amino acid complement was rather different for each species, and more similar between *P. suberosa* and *P. misera* as compared to *P. caerulea* ; the last species is included in a different subgenus than the one of the other two (Feuillet and MacDougal 2004). Difference in amino acid composition was also found in EF nectar of 8 *Inga* species (Koptur 1994), even species with versus species without amino acids, but not finding consistency with taxonomy.

### 4.4 EF Nectar Feeders Occurrence and Behavior

The visitors to EF nectaries registered so far belong to several orders and families of arthropods (Koptur 1992; Jolivet 1998) but members of Lonchaeidae, Berytidae, and Tingidae are recorded at passion vines EF nectaries for the first time.

The EF nectar feeders were not specific of the plant species investigated; though, the differences in visitor composition could be due to insect local abundance or alternative resource availability. Species of *Camponotus* and *Crematogaster*, as well as ants included in different genera, were reported as consumers of EF nectar of other *Passiflora* spp. (McLain 1983; Lanza 1988; Christensen and Olesen 1998; Apple and Feener Jr 2001; Monteiro Macedo et al. 2021; Ribeiro Soares et al. 2025). Different ant species visited *Calathea ovadensis* at different sites in Mexico mountains (Horwitz and Schemske 1984 cited in Howe and Westley 1988). The lack of appearance of *C. iheringii* on certain *Passiflora* species is not explained by their distribution range, as this ant is widely distributed in Argentina (Kusnezov 1978). In studies concerning sympatric *Passiflora* species, not all passion vine species were visited by the same ant species (Christensen and Olesen 1998), even ants did not patrol all the time (Smiley 1986) nor all the individual plants of a single species (Christensen and Olesen 1998). *Polybia occidentalis scutellaris* visited *P. misera* in Merlo probably because of the proximity of the nest, but was absent in C.U.; this wasp was abundant in Buenos Aires province (Llano 1959) but now it might be restricted to wilder areas.

The repetitive licking on a single nectary of *C. mus* individuals suggests that they did not take all the nectar at a time and the antennation between workers moving in opposite directions along the stems suggests recruitment to accurate places.

As it was observed for *C. iheringii* on *P. misera* here, some ant workers occasionally cover small liquid sources near the nest (Hölldobler and Willson 1990). Barriers built with debris around EF nectaries of *Passiflora auriculata* by *Crematogaster* ants had been interpreted as a resource hiding strategy that excludes competing family relatives (Eskildsen et al. 2001).

Considering their behavior, some EF nectar visitors could play a defensive role (*C. mus, C. iheringii* and the wasps); it rests to quantify if they influence the herbivory rate. This seems to depend on relative densities of every interaction partner; ants chased away or bothered chrysomelids on *P. mooreana* in Charata (Chaco province, Argentina) (Amela García 1994) where these beetles were more abundant than in the localities involved in this study. Ants in a threatening position as described above have been reported by Hocking (1975) and members of the Camponotini exhibit their abdomen as a ready‐to‐use chemical weapon when defending a food source (Silvestre et al. 2003). In relation to *C. iheringii* , species of this genus eat heliconian eggs (Benson et al. 1975), though not observed here. By one hand, the wasp *Polistes* sp. is likely to be more effective, as it just preyed on the lepidopteran larvae, in contrast to the simple bothering done by the ants, but on the other hand, their abundance and frequency were much lesser than the permanently patrolling ants. A negative impact of predaceous *Polistes* spp. wasps on heliconiine caterpillars, in contrast to ants, has been reported in *P. biflora* (Ward 2002). Anyway, defense of *Passiflora* spp. by wasps might not be so established as these insects reject unpalatable prey after several probes (Schowalter 2006), if *Passiflora* caterpillars are unpalatable and/or toxic, which has not been probed yet but it is highly probable, as sometimes dead bitten caterpillars are found on the plants. Anyway, ants can defend plants just by annoyance to herbivores (de la Fuente and Marquis 1999).

Not all the EF nectary feeders registered are predators or aggressive (e.g., flies, bees, bugs), so they should be considered as extrafloral nectar thieves, as has been done for flies (Heil and Linsenmair 2004) and moths (Sourakov 2008). Moreover, the Tinginae, besides licking nectaries, suck phloem sap and the Lonchaeidae flies seem to develop inside passion vine blossoms (Amela García 1999). Ants chased away several fly species that lick the EF nectaries of *P. mooreana* (Amela García 1994). This action would additionally benefit the plant, as most of the EF nectar would be assured for the ants, that is, the putative and most constant plant herbivore defenders.

### 4.5 EF Nectar Feeders Preferences

The attractiveness of sugars to tested ant species increases with their concentration (Koptur 1992) and, thus, the protective effect (Fagundes et al. 2017); sucrose is preferred to others (Blüthgen and Fiedler 2004b), or sucrose or glucose over fructose (Koptur 1992), but *Pseudomirmex* symbiotic ants are fond of hexose nectars (Heil et al. 2005). The optimum sucrose intake rate ranged from 40%–50% w/w for three ant species (references in Nicolson 2007), being that of *C. mus* of 43% w/w (Josens et al. 1998), but this may widen with starvation (Josens and Roces 2000 cited in Nicolson 2007). Some ants, including *Passiflora* spp. EF nectary visitors, prefer sugar solutions with certain amino acids instead of sugar‐only solutions (Lanza and Krauss 1984), the amino acid‐containing artificial mimic solutions against the sugar‐only ones (Lanza 1988) or the richer amino acid mimic (Lanza et al. 1993); but not all the ants are more attracted to richer amino acid solutions (Lanza et al. 1993) and different species choose different amino acids (Blüthgen and Fiedler 2004b), even differ in the nutritional profitability from a single food source (Fisher et al. 1990).

Regarding flies, *Ceratitis capitata* optimum sucrose intake was at 24% w/w (Warburg and Galun 1992 cited in Nicolson 2007). This value is quite similar to the EF nectar concentration of *P. caerulea* and *P. misera* , whose nectars were licked by Lonchaeidae members, other fruit fly (ICA 2012). The western cherry fly feeding time increased with sucrose concentration (Yee 2003), but the concentration and volume of the solution chosen by *Drosophila melanogaster* depended on starving conditions (Edgecomb et al. 1994 cited in Nicolson 2007). Flesh flies preferred sugar‐amino acid nectar or amino acid‐only nectar mimics over sugar‐only ones (Rathman et al. 1990); instead, the Caribbean fruit fly did not consume differently sucrose only versus sucrose plus amino acid solutions in no choice tests, but when the amino acids were changed, the amino acid solutions were preferred to the sucrose alone (Nigg et al. 2007).

Considering wasps, sucrose‐based baits were more efficient than protein‐based ones (Spurr 1996). These insects seem to exploit any available source of concentrated sugar, but certain species may also require proteinaceous food, especially when sugar sources are scarce (Evans and Eberhard 1970).

So, generally, the sources with a major amino acid content are more attractive to ants (Blüthgen et al. 2004) and to other insects (Rathman et al. 1990). This was reflected in the greater species richness of EF nectary visitors at least in *P. misera* , which, together with *P. suberosa* , exhibited a significantly greater amino acid concentration than *P. caerulea* .

The presence of ants on certain resources may not only reflect preferences but also competition, where the dominant species monopolizes the richer amino acid sources (Blüthgen and Fiedler 2004a) and even restricts to individual plants or certain leaves (Mody and Linsenmair 2003). Different species of ants chased away each other from the EF nectaries of *P. mooreana* (M. T. Amela García unpublished data). Some Crematogastrini species are part of the dominant groups in ant mosaics (Fernández 2003a). *Crematogaster* spp. are highly territorial on food resources (Silvestre et al. 2003); on the contrary, *Camponotus* species are usually opportunistic. This might explain why *P. suberosa* sugar sources (EF nectar and coccid honeydew) were used almost exclusively by *C. iheringii* , while *C. mus* patrolled *P. misera* in the same site; even more, some workers of *C. iheringii* licked sometimes the nectaries of *P. misera* while *C. mus* only licked the teratological nectaries of *P. suberosa* .

Due to the similarities of preferences between ants, wasps and flies for sugar and amino acid sources, their visits to the nectaries seem to be determined more by abundance of each visitor group at each site and/or competitive interactions rather than by special requirements of the various visitors.

### 4.6 Herbivore Specificity

Most of the herbivores recorded on the investigated *Passiflora* species are more or less specific of this genus. *D. vanillae maculosa* only feeds on passion vines (Posada Ochoa 1989; Pastrana 2004) and the specificity of the Heliconiinae caterpillars on Passifloraceae has been studied in detail (Benson et al. 1975; Turner 1981 and cites therein, among others). *C. melanoptera* adults and *Systena* sp. feed on several *Passiflora* species (Amela García 1999; Cordo et al. 2004), as other flea beetles do (Smiley 1982; Pemberton 1989), which seem to accumulate their toxic compounds (Smiley 2013). *H. histrio* has been recorded exclusively on *Passiflora* spp. (Di Iorio, pers. com.) exhibiting tolerance (Baldin and Boica 1999) or preference (Caetano et al. 2000) for certain species, while coreids of other genera also suck *Passiflora* spp. (Posada Ochoa 1989). Only *Gargaphia lunulata* has a wider host range (Ojeda et al. 2017). This bug only attacked the *P. misera* specimens inside the greenhouse during the course of this study, though there are reports of infestation of *P. caerulea* in Buenos Aires (Ajmat et al. 2003); the lack of infestation of *P. caerulea* and *P. misera* in Merlo could be due to absence of these bugs in that locality; besides, it seems to attack plants under greenhouse conditions more intensely, as the beans cultivated by Ojeda et al. (2017). Contrarily, *C. melanoptera* never appeared in the greenhouse, in spite it was present on the plants in the surrounding campus. The extrafloral nectar thieving Lonchaeids observed on *P. caerulea* have probably some degree of specifity with this species, as passion vines are hosts of Lonchaeidae members (Steyskal 1980; Posada Ochoa 1989; ICA 2012). Relying on the exclusive/relative specificity of the taxa here registered as well as their behavior, their interactions with ant and/or wasp guards acquire more relevance, so future investigations are needed to determine in what circumstances (mainly concerning relative abundances) ants and wasps may constitute an antiherbivore defense in these passion vines.

### 4.7 Future Perspectives

This research explored three *Passiflora* species in depth; tough, considering the more than 600 species in the genus recognized so far (Ocampo Pérez and Coppens d'Eeckenbrugge 2017), and just about 20 dealt with until now concerning EF nectar and its mediated interactions, future studies are needed to characterize extrafloral nectary morphology, nectar composition, and insect associations in other co‐generic members, which could contribute to understanding multi‐trophic interrelationships to a broader extent.

## 5 Conclusion

Through this study, some differences in nectar profiles, especially amino acids, were found between *Passiflora* species included in different subgenera, thus envisioning its relevance in taxonomy. Extrafloral nectary morphology and function, either when producing nectar as well as afterwards, have been described, with the interplay of various arthropod actors, which involved butterfly egg mimics, incidental domatia and attraction to ants and wasps, all putative anti‐herbivore defense traits acting at various trophic levels, including nectar thieves. Further studies, involving other *Passiflora* species and larger laborious‐to‐collect samples, will help to comprehend the adaptations of this genus.

## Author Contributions

**M. T. Amela García:** conceptualization (lead), data curation (lead), formal analysis (equal), funding acquisition (lead), investigation (lead), methodology (equal), project administration (lead), resources (lead), software (equal), supervision (equal), validation (equal), visualization (equal), writing – original draft (lead), writing – review and editing (equal). **L. Rodríguez‐Planes:** conceptualization (supporting), data curation (supporting), formal analysis (supporting), methodology (supporting), software (supporting), validation (supporting), visualization (supporting), writing – review and editing (supporting). **G. Gottsberger:** conceptualization (supporting), data curation (supporting), formal analysis (supporting), methodology (equal), resources (equal), supervision (supporting), validation (supporting), visualization (supporting), writing – review and editing (supporting).

## Funding

This work was supported by ANPCyT (Agencia Nacional de Promoción Científica y Tecnológica), PICT 32866.

## Conflicts of Interest

The authors declare no conflicts of interest.

## Supporting information


**Data S1:** aaextrafl.
**Data S2:** caerulea sugars.
**Data S3:** EFN_secretion.
**Data S4:** EFN_sugars.
**Data S5:** vol_extrafl.

## Data Availability

All the required data are uploaded as Supporting Information.
